# Performance Comparison of NB-Fi, Sigfox, and LoRaWAN

**DOI:** 10.3390/s22249633

**Published:** 2022-12-08

**Authors:** Polina Levchenko, Dmitry Bankov, Evgeny Khorov, Andrey Lyakhov

**Affiliations:** Institute for Information Transmission Problems of the Russian Academy of Sciences, 127051 Moscow, Russia

**Keywords:** NB-Fi, Sigfox, LoRaWAN, LPWAN, ultranarrow band, IoT, sensor networks, performance evaluation

## Abstract

LPWANs are a promising solution for wireless sensor networks. To compete with such widespread technologies as LoRaWAN and Sigfox, recently a new LPWAN technology called NB-Fi has been developed. In a short time, many NB-Fi networks have been deployed in various countries. Although NB-Fi, Sigfox, and LoRaWAN have been designed for similar applications, they implement different approaches. However, no detailed comparisons of them are present in academic literature. This paper aims to fill this gap by analyzing and comparing NB-Fi, Sigfox, and LoRaWAN focusing on performance evaluation results rather than just nominal parameters declared by the developers. Specifically, the paper evaluates the packet loss rate, packet error rate, and average delay in these networks in different scenarios. The results are used to provide guidelines to decide which technology to use under which conditions. Specifically, Sigfox performs best in scenarios when devices transmit small pieces of data without repetitions and acknowledgments, and LoRaWAN is the most reliable for transmitting bigger pieces of data, while NB-Fi is best suited for acknowledged transmissions of small pieces of data.

## 1. Introduction

The Internet of Things (IoT) is rapidly evolving. According to recent studies [1], 27 billion devices will be connected to the IoT by 2025, many of which will require low energy consumption and a wide area of connectivity. Low-power wide-area networks (LPWANs) satisfy these requirements and serve large numbers of devices over long distances with relatively low energy consumption. One of the most popular LPWAN technologies is LoRaWAN: LoRaWAN networks have been deployed in more than 170 countries [2]. Another popular LPWAN technology is Sigfox, which is used in more than 70 countries [3]. NB-Fi is a rather new LPWAN technology, but in a short time, NB-Fi networks have been deployed in Argentina [4], India [5], France [6], Kazakhstan [7], Moldova [8], Russia [9], and Serbia [10].

Although NB-Fi is designed for similar scenarios as LoRaWAN and Sigfox, it uses different approaches. By now, no detailed comparisons of NB-Fi, Sigfox, and LoRaWAN exist in scientific literature, except for a few surveys that compare their nominal operational parameters such as modulation and coding schemes and corresponding bitrates. While these parameters allow us to calculate only the PHY layer nominal throughput for “point-to-point” connections [11,12], the end-users are more interested in the overall performance of the systems with thousands of devices. Unfortunately, the performance at the upper layers for such large networks significantly depends on the overhead induced by packet headers and transmission protocols, inter-packet gaps, channel access procedure (all the mentioned technologies use random channel access), and the approaches to resolve collisions during random access. As a result, it is not clear which technology shall be selected for a particular use case.

The goal of this paper is to compare the performance of NB-Fi, Sigfox, and LoRaWAN in various scenarios to determine which technology and conditions serve the traffic more efficiently. Specifically, we compare how the packet loss rate (PLR), packet error rate (PER), and the average delay depend on the load if the amount of available frequency resources is the same. The obtained values of PLR and PER are directly linked to the network capacity, which is an essential metric for LPWANs and determines the maximal number of devices that can concurrently operate in a network with an acceptable service.

The rest of the paper is organized as follows: In Section 2, we review related papers. Section 3 introduces NB-Fi, Sigfox, and LoRaWAN. Section 4 describes the considered scenario and the problem statement. Section 5 presents and discusses numerical results. The conclusions are given in Section 6.

## 2. Related Works

LPWAN technologies have attracted much attention in the literature. Many surveys evaluate and compare nominal performance indices of various LPWAN technologies provided by their specifications. Notably, these values may significantly differ from the system-level performance of large sensor networks. For example, in [12], the authors describe a general IoT infrastructure and industrial IoT (IIoT) as a significant part of it. The authors describe technologies that can be used for IIoT, such as NB-IoT and LTE-M, which use licensed frequency bands, and LoRa, Sigfox, and NB-Fi, which use unlicensed frequencies. Even though NB-Fi, Sigfox, and LoRaWAN are mentioned as promising solutions for IoT, the efficiency of their networks is not studied.

In [11], the authors describe such LPWAN technologies as LoRaWAN, Sigfox, NB-Fi, Nwave, and RPMA. They provide nominal characteristics of these technologies, such as the maximal data rate, transmission range, number of devices connected to a base station (BS), etc., while the real network performance requires more detailed study in various scenarios.

Some authors design analytical models, simulations, or testbeds to evaluate the system-level performance of various technologies. For example, in [13], the authors briefly analyze the channel access method in NB-Fi and point out the factors that reduce the packet loss ratio (PLR) and delays. Ref. [14] designs a mathematical model of a Wi-Fi HaLow sensor network. In [15,16], the authors analyze the performance of large-scale Sigfox and LoRaWAN networks, respectively. As they consider different scenarios, their results do not allow for comparing the performance of various technologies under the same conditions.

The channel access method in these technologies is a kind of ALOHA, which is asynchronous both in time and in the frequency domain in the NB-Fi and Sigfox protocols. In LoRaWAN, the channel access is asynchronous in time but slotted in frequency. Despite the similarity of the channel access, the peculiarities of each technology do not allow the development of a simple one-size-fits-all mathematical model, though many approaches have been attempted.

For example, the authors of [17] use stochastic geometry methods to describe the time-frequency interference and show how to find the outage probability and throughput for Sigfox and LoRaWAN networks with this model. However, the developed model describes only the case when all frames have the same duration and width in the frequency domain, so this model cannot be used for NB-Fi where devices can use different bitrates, and the frames have variable duration and width. In addition, in the considered model, devices generate saturated traffic, which is not a typical scenario for sensor networks.

Many papers compare various technologies with simulation. For example, Ref. [18] evaluates PER of Sigfox, LoRaWAN, and NB-IoT. However, the authors consider slotted simulation time, where one slot equals the duration of one frame, but since both Sigfox and LoRaWAN use asynchronous random access in the time domain, results from this paper cannot be used to accurately compare the performance of the considered LPWAN technologies.

Ref. [19] considers how PLR depends on the number of sensors in Sigfox and LoRaWAN networks. The authors conclude that both technologies are promising for IoT; however, they do not explicitly compare the performance of the considered networks.

In ref. [20], the authors provide an experimental comparative evaluation of LoRaWAN and Sigfox based on coverage and energy-efficiency test performance. The authors provide a thorough evaluation of networks’ coverage by measuring the Received Signal Strength Indicator (RSSI) and calculating the packet delivery rate (PDR) in rural and urban environments. The results were obtained experimentally for 11 static measurement points. However, this paper considers only the problem of signal propagation but omits the effects on the network performance of the collisions caused by random access due to many sensors operating in the network. Moreover, the authors allocate each network with the channel resources that would maximize the network’s coverage, but these resources are different for Sigfox and LoRaWAN.

The analyses of prior art bring us to the following conclusion. The comparison of various LPWAN technologies is very important both from scientific and practical points of view, which is confirmed by many papers on this topic. However, because of its novelty, NB-Fi is typically not considered in such studies. This paper fills this gap and compares NB-Fi with Sigfox and LoRaWAN both (i) by their nominal parameters and (ii) by the system-level performance in various scenarios, in order to determine the best-suited technology for each scenario.

## 3. An Overview of NB-Fi, Sigfox, and LoRaWAN

NB-Fi, Sigfox, and LoRaWAN networks operate in unlicensed ISM radio bands that impose limitations on the emitted power. For example, in Russia, NB-Fi, Sigfox, and LoRaWAN networks use the band of 868.7–869.2 MHz with the limit of 100 mW for emitted power.

A typical NB-Fi, Sigfox, and LoRaWAN network has a “star of stars” topology and consists of end-devices (further related to as *sensors*), base stations (BSs), and a server. In the considered LPWANs, BSs are connected with the server via a broadband link or the server may be implemented on the BS. Sensors communicate with the server via BSs using wireless NB-Fi, Sigfox, or LoRaWAN links, while BSs act as transparent retransmitters between the sensors and the server. Sensors are not associated with a single BS, so any BS that receives a frame from a sensor redirects it to the server, while, in the reverse direction, the server chooses which BS transmits a frame to the sensor. NB-Fi specification is centered around local regulations in European countries, while Sigfox [21] and LoRaWAN specifications [22,23] indicate radio parameter values for a set of countries. Therefore, in this paper, we consider European operational mode, since all of them—Sigfox, NB-Fi, and LoRaWAN—can operate in the same European band.

Let us initially compare the nominal parameters of these technologies, i.e., their modulation schemes, frame formats, channel access rules, and operation modes.

### 3.1. Modulation

#### 3.1.1. NB-Fi

NB-Fi sensors communicate with BSs using binary phase-shift keying (BPSK) or differential binary phase-shift keying (DBPSK) in the downlink and DBPSK in the uplink.

**Figure 1:**
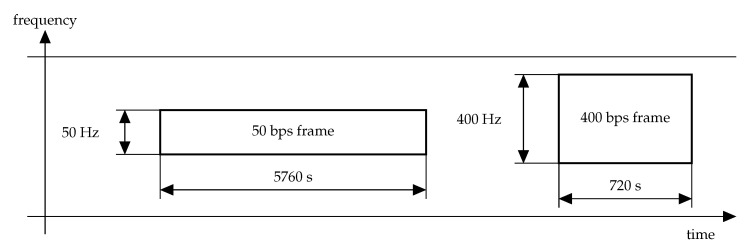
Different frame widths and durations in NB-Fi.

In NB-Fi, frames can be transmitted at four different rates: 50, 400, 3200, or 25,600 bps. The rate affects the frame duration and the bandwidth used for the frame transmission (see Figure 1). As the use of a narrow band increases the power spectral density, the lower is the rate, the higher can be the transmission range, i.e., the maximal distance between the transmitter and the receiver at which the communication is still reliable.

The receiver sensitivity is calculated as

(1) $$S_{crit}=10\log_{10}kT\Delta f+N_{base}+SNR_{BER},$$

where $N_{base}$ is the input noise, $SNR_{BER}$ is the signal-to-noise ratio (SNR) corresponding to the given bit error rate, *k* is the Boltzmann constant, *T* is the temperature, and Δf is the signal frequency band. Therefore, for the values given in the standard [24]: T=290K, $BER=10^{-5}$, $SNR_{BER}=5$, $N_{base}=2$, we obtain the values of sensitivity for different bitrates, shown in Table 1.

#### 3.1.2. Sigfox

Sigfox implements the DBPSK modulation in the uplink and Gaussian Frequency Shift Keying (GFSK) in the downlink.

In Sigfox, sensors may select the symbol rate on a per-message basis and according to regional profile permissions. For example, the European configuration allows two bitrates: 100 and 600 bps. Similarly to NB-Fi, lower rate results in lower bandwidths used for the frame transmission, lower maximal power at which the communication is possible, and wider maximal transmission range, but higher frame durations.

According to the specification [21], the downlink sensitivity equals −126 dBm, while the uplink sensitivity specified on Sigfox support’s official website [27] equals −135 dBm.

#### 3.1.3. LoRaWAN

LoRaWAN sensors communicate with BSs using the LoRa technology, which is based on the Chirp-Spread Spectrum (CSS) modulation. In LoRa, the signal continuously varies in frequency within window BW around the central frequency $f_{c}$. It means that the frequency starts with one of the $2^{SF}$ values within $\left[f_{c}-\frac{BW}{2};f_{c}+\frac{BW}{2}\right]$, where SF is the spreading factor, which defines the symbol duration and the number of bits per symbol. Then, the frequency increases until $f_{c}+\frac{BW}{2}$, drops to the minimum $f_{c}-\frac{BW}{2}$ and grows until reaching the initial value. The key feature of the LoRa modulation is that frames transmitted with different SFs can be received and decoded simultaneously [23].

The total bitrate can be calculated as follows [28]:

(2) $$R_{b}=\frac{SF\times CR}{\left[\frac{2^{SF}}{BW}\right]},$$

where CR is the rate of Hamming code and can be $\frac{4}{5},\frac{4}{6},\frac{4}{7},\frac{4}{8}$ [22].

Therefore, the bitrate depends on the BW, SF, and CR. Higher values of SF result in lower bitrates; however, signals can be received at higher receiver sensitivities. Table 1 lists bitrates, SF, critical receiver sensitivities, and CR for BW=125 kHz.

### 3.2. Frame Format

#### 3.2.1. NB-Fi

**Figure 2:**
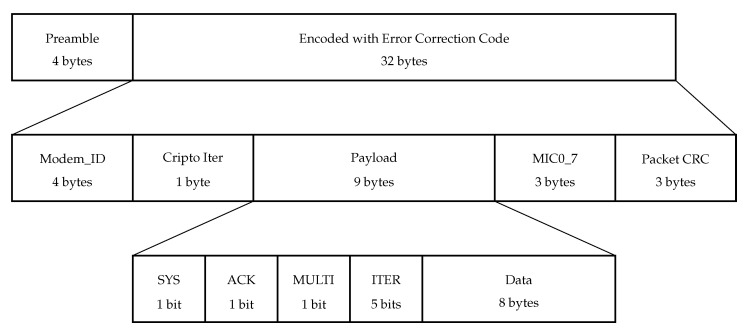
Uplink frame structure in NB-Fi.

The uplink frame structure in NB-Fi is shown in Figure 2. It starts with a four-byte long preamble that serves for synchronization. It is fixed and equals 0x97157A6F in hexadecimal form.

The preamble is followed by data encoded with an error correction code, which is either a convolutional code or a non-systematic polar core [29] with the code rate of $\frac{5}{8}$.

The encoder input is 20-byte long and consists of the following fields:

*Modem_ID:* a 32-bit sensor identifier.
*Crypto Iter:* 8 least significant bits of the cryptoiterator, which is a 32-bit counter stored at the device and incremented by one for every transmitted frame. NB-Fi devices use the Magma [30] symmetric key block cipher to produce security keys and encrypt the payload. When Crypto Iter makes a full turn, devices update the security keys. Devices also use the cryptoiterator to produce the start value for payload encryption.
*Payload:* a 9-byte field with encrypted payload. It contains a transport layer packet, which consists of a header (1 byte) and the upper layer payload (8 bytes). The header contains four fields:
  - SYS: a flag that identifies service frames;
  - ACK: a flag that informs that the data frame requires acknowledgment;
  - MULTI: a flag that is needed for block transmissions and informs that the frame is not the last in the block;
  - ITER: a 5-bit field that is used for numerating frames in a block and can take values from 0 to 31.
*MIC0_7:* 3 least significant bytes of the message authentication code (MAC) which is also calculated using the Magma algorithm in Encrypt-then-MAC mode; MIC0_7 is used to make sure that the payload has not been changed. In addition, as described further, MIC0_7 is used to calculate the central frequency of the transmission.
*Packet CRC:* Three least significant bytes of the CRC32 checksum calculated from Modem_ID, Crypto Iter, and Payload.

**Figure 3:**
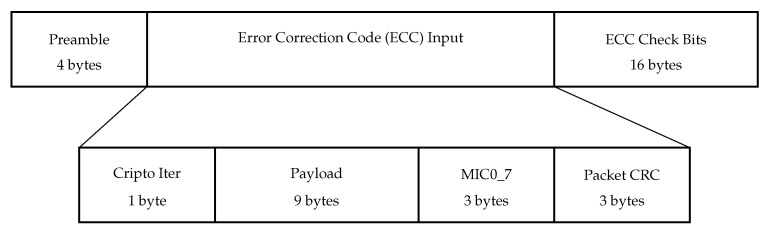
Downlink frame structure in NB-Fi.

Figure 3 shows the downlink frame structure in NB-Fi. As opposed to the uplink frame, the downlink frame does not contain Modem_ID, and the preamble is not fixed but is calculated for every transmission according to an algorithm based on the iterative pseudorandom number generator, where the receiver’s Modem_ID is the starting position of the generator. The preamble is followed by the Crypto Iter, Payload, Data Authentication Code, and Packet CRC, which are similar to the fields in the uplink frame. These four fields are used as input to the Zigzag error correction code [31] with the code rate of $\frac{1}{2}$. The downlink frame ends with the control bits produced by the Zigzag encoder.

#### 3.2.2. Sigfox

**Figure 4:**
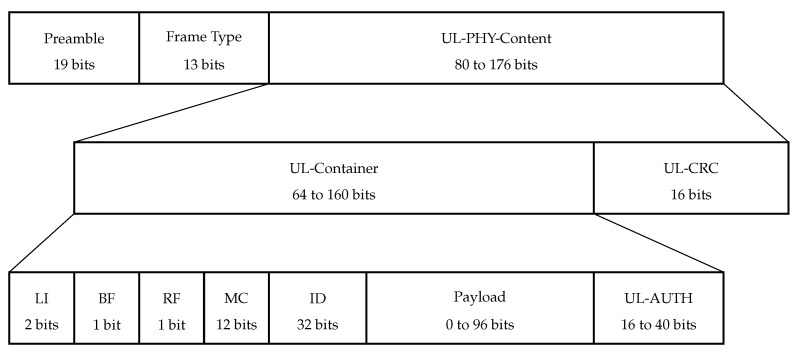
Uplink frame structure in Sigfox.

Sigfox uplink frame structure is shown in Figure 4. It begins with a fixed 19-bit preamble: 101 0101 0101 0101 0101.

The preamble is followed by the Frame Type field (FT) which is 13 bits long and encodes UL-Container length, message type, and frame emission rank.

The next field is UL-PHY-Content which is the result of encoding UL-Container and UL-CRC with a convolutional code with code rate $\frac{1}{3}$.

The UL-CRC field is 16 bits long and is required to evaluate the integrity of the frame.

The UL-Container is 64 to 160 bits long and consists of the following fields:

*Length Indicator (LI):* a 2-bit field. Its value depends on Payload and UL-AUTH;
*Bidirectional Flag (BF):* a 1-bit field, set to 0 for U-procedure and to 1 for B-procedure;
*Repeated Flag (RF):* a 1-bit field. Its value is set to 0;
*Message Counter (MC):* a 12-bit field. The sensor increments the message counter by 1 every time a message is sent in the uplink;
*Identifier (ID):* a 32-bit field, which consists of sensor identifier bytes loaded in reverse order;
*Payload:* an up to 96-bit field containing the message content;
*UL-AUTH:* 2 to 5 most significant bytes of the AES-based transformation of LI, BF, RF, MC, ID, and Payload.

**Figure 5:**
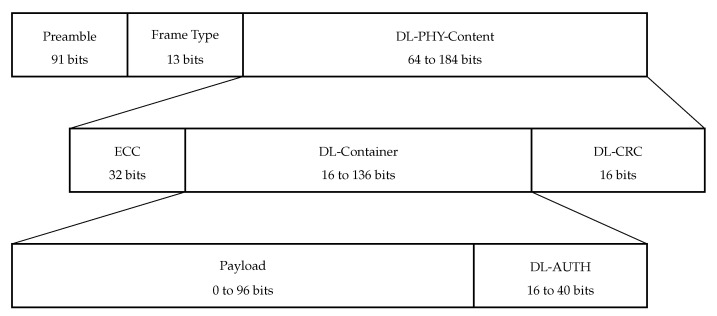
Downlink frame structure in Sigfox.

Sigfox downlink frame structure is shown in Figure 5. The preamble in the downlink frame is 91 bits long as opposed to the 19-bit long field in the uplink frame. The DL-PHY-Content is the result of whitening by XOR-ing ECC, DL-Container, and DL-CRC with a pseudo-random sequence. Error correction code (ECC) is a 32-bit field and is the result of the implementation of BCH15-11 Error Correction Codes over the concatenation of DL-CONTAINER and DL-CRC fields. The next field is the DL-Container, which consists of Payload (0 to 96 bits) and DL-AUTH (2 most significant bytes of the AES-based transformation of Payload). The DL-Container is followed by DL-CRC, which is required to evaluate the integrity of the frame.

#### 3.2.3. LoRaWAN

**Figure 6:**
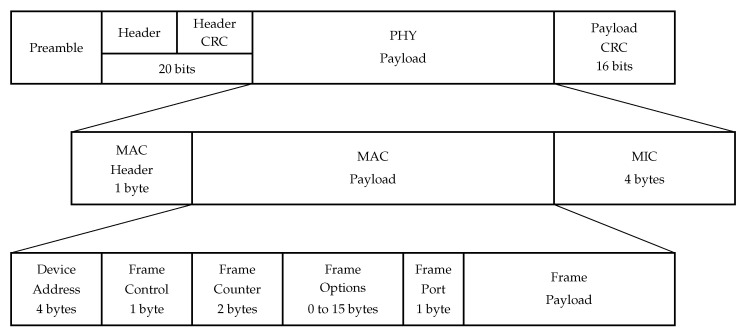
Uplink frame structure in LoRaWAN.

The uplink frame structure is shown in Figure 6. It begins with a preamble, which allows the receiver device to synchronize and defines the frame modulation scheme since it is modulated with the same spreading factor as the rest of the frame. Typically, the preamble duration is 12.25 $T_{s}$, where $T_{s}$ is the symbol duration.

The preamble is followed by a PHY Header and Header CRC fields, which are 20 bits long together and are encoded with the most reliable code rate of $\frac{4}{8}$. The Header field contains information about the code rate, payload length, and whether the Payload CRC field is present in the frame (downlink frames do not contain the Payload CRC field).

The frame duration is $T_{frame}=\left(n+12.25\right)T_{s}$, where *n* is the number of symbols needed to transmit the PHY Payload and the PHY Header and is calculated as follows [32]:

(3) $$8+max\left(\left[\frac{8PL-4SF+28+16\;\cdot \;[CRCispresent]}{CR(SF-2DE)}\right],0\right).$$

PL is the payload length in bytes, SF is the spreading factor, CR is the code rate, and CRCispresent equals 1 if the PHY Header CRC is present in the frame, which means that it is the uplink frame, and 0, otherwise; DE is 1 if the low data rate optimization is enabled and 0 otherwise. Low data rate optimization is an option that can be enabled in LoRa transmitters to improve the robustness of transmission to variations in frequency.

The next field is the PHY Payload. It starts with a MAC Header, which is 1 byte long and contains information about protocol version, message type (data/management, uplink/downlink frame), whether the message should be acknowledged, or if it is a vendor-specific message.

MAC Header is followed by a Frame Header which contains the following information:

*Device Address:* a 4 byte field. The first two bytes identify the network; other bits are assigned dynamically and identify the sensor in the network;
*Frame Control:* a 1-byte field, used for network control information, such as whether to use the data rate specified by the BS for uplink transmission, whether this message is an acknowledgment of the previous message, whether the BS has more data for the sensor;
*Frame Counter:* sequence number;
*Frame Options:* a 0 to 15 bytes long field, which is used for changing data rate or transmission power, validating connection, etc.;
*Frame Port:* allows for distinguishing several flows between a sensor and a BS. If it is equal to 0, then the frame contains MAC commands instead of user data.

The next field is MIC, which is used as a digital signature of the message.

The overall size of the PHY Payload field is 13+FP+FOps bytes, where FP is the Frame Payload length and FOps is the Frame Options length. Therefore, if a BS or a sensor wants to send an empty message (i.e., to acknowledge frame reception), they will have to send 13 bytes of payload in it, which leads to significant overhead. The maximal Frame Payload length and bitrate depend on the SF, used to transmit the frame. Table 2 lists maximal and minimal values of frame durations in the uplink and downlink $T_{up}^{max}$, $T_{up}^{min}$, $T_{down}^{max}$, $T_{down}^{min}$, and the maximal values of Frame Payload PL depending on the SF. Note that, if a BS does not have any data to transmit and simply wants to acknowledge the reception, it transmits a frame in the downlink without any payload, which means that the duration of such frame equals $T_{down}^{min}$.

In LoRaWAN, the downlink frame structure is identical to the uplink frame except for the Header CRC field, which is omitted in the downlink frame.

### 3.3. Channel Access and Operation Modes

#### 3.3.1. NB-Fi

In NB-Fi, the operating frequency band can be configured by the operator and is at least 51.2 kHz in the uplink and 102.4 kHz in the downlink. The uplink and downlink frequency bands do not intersect.

For uplink transmissions, sensors calculate the central frequency as a function of bitrate, Modem_ID, and MIC0_7. MIC0_7 with high probability changes for each transmission; therefore, central frequency in uplink is different for each transmission, including retransmission and block transmission. For downlink transmissions, the server uses a central frequency which is a function of bitrate and Modem_ID. Therefore, the server always transmits frames intended for one sensor at the same central frequency.

NB-Fi sensors can operate in three modes. By default, the sensors operate in the discontinuous RX mode (DRX). In this mode, the sensors transmit data if needed and listen to the downlink channel for a short period of time after the end of their transmission. The server buffers all data that have to be transmitted to each sensor and transmits the data during the time interval, when the appropriate sensor is listening to the channel.

**Figure 7:**
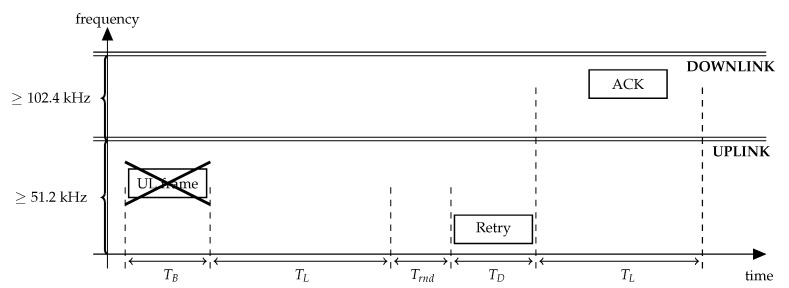
DRX transmission procedure in NB-Fi (here, the first transmission attempt is unsuccessful).

Let us consider the transmission procedure of an NB-Fi sensor working in the DRX mode, illustrated in Figure 7. $T_{B}$ seconds after the start of the frame transmission, the sensor listens to the downlink channel for $T_{L}$ seconds. If the sensor does not receive an acknowledgment, intended for it, it makes a retry after a random backoff time uniformly distributed within the retry window $T_{rnd}$. The sensor retransmits the frame until it receives an acknowledgment or exceeds a configurable retry limit. Table 3 lists $T_{B}$, $T_{L}$, and $T_{rnd}$ values.

The second mode, called No RX, supports only one-way communications from sensors to BS. In this mode, the sensors transmit data if needed. Otherwise, they stay in the “sleep” state with their radio switched off.

In the last mode, called continuous RX mode (CRX), the sensors always listen to the channel and can transmit and receive data at any time. Most of the devices in sensor networks are battery-powered and, therefore, do not use this mode. Thus, we do not consider the CRX mode in this paper.

#### 3.3.2. Sigfox

The operating frequency band in Sigfox networks is 192 kHz wide. To transmit a frame, Sigfox sensors can use one of the two modes: U-procedure and B-procedure. With U-procedure, sensors send their frames in the uplink with no onward downlink frame. With B-procedure, sensors request a response to their uplink frame and thus start a bidirectional procedure. The Sigfox operator imposes a limit on the downlink transmission equal to four frames per day. It means that downlink is not intended for constant use. Therefore, we do not consider B-procedure in this paper. The sensors may send a frame once or three times, which improves transmission reliability. Sigfox rules allow for creating and transmitting one (single frame procedure) or three frames during one procedure (multiple frame procedure). In case three messages are transmitted, they must be identical, except for frame type (FT) (see Section 3.2) and convolution code.

In any case, the first transmission attempt occurs at a random central frequency $f_{1}$ within the uplink channel. If the sensor transmits the frame only once, it waits for $T_{P}\geq 10$ ms after the transmission before it can initiate a new one. Such an interval is introduced to simplify the detection and reception of uplink frames by the BS.

**Figure 8:**
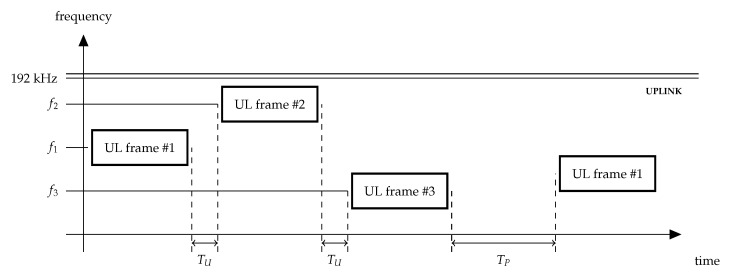
Multiple frames U-procedure in Sigfox.

Otherwise, see Figure 8, the sensor repeats the transmission twice at random central frequencies $f_{2}$ and $f_{3}$, each time waiting for $10\leq T_{U}\leq 2000$ ms after the previous transmission. The sensor completes the transmission procedure $T_{P}\geq 10$ ms after it finishes the third transmission.

If the sensor needs to transmit several frames in a row, so-called block transmission, it accesses the channel independently for each frame. Moreover, the sensor may switch between one and three transmission attempts for each frame.

#### 3.3.3. LoRaWAN

In LoRaWAN networks, the operating channels can be 125 kHz, 250 kHz, or 500 kHz wide. A LoRaWAN operator divides the available spectrum into several channels. The number of channels depends on regional restrictions and network options. Some channels are used for data transmission (hereafter called the main channels), some channels are reserved for join requests sent by sensors, wishing to connect to the server, and one channel is used for downlink responses by the BSs (hereafter called downlink channel).

LoRaWAN sensors can operate in three modes called Classes A, B, and C. Class A is the default operation mode supported by all LoRaWAN devices. Class A sensors are capable of bi-directional communications. They transmit data if needed and can receive data only shortly after transmitting. Downlink communications from the server at any other time will have to wait until the next uplink transmission.

**Figure 9:**
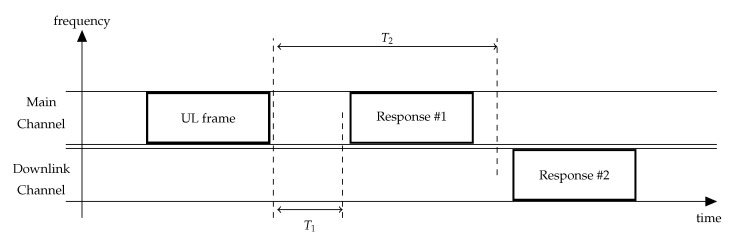
Class A transmission procedure in LoRaWAN.

Let us consider a Class A sensor. Figure 9 illustrates its transmission procedure. If the sensor has some data to transmit, it randomly selects one of the main channels and transmits the frame. Note that, during the transmission, the frame occupies the whole channel. $T_{1}$ seconds after the transmission, the sensor listens to the same uplink channel where the data were transmitted for a short time. By default, $T_{1}=1$ s. If the sensor receives a downlink frame intended for it and the MIC field checks out, then the sensor does not open the second receive window. Otherwise, the second receive window is opened $T_{2}=T_{1}+1$ s after the end of uplink transmission. During this receive window, the sensor listens to the downlink channel. If a preamble is detected during one of the receive windows, the sensor does not close the receive window until the downlink frame is demodulated. If the sensor does not receive any of the acknowledgments, it retransmits the frame after a random delay within the retry window [1;3] s. The sensor retransmits the frame until it receives an acknowledgment or exceeds a configurable retry limit. The receive window duration is configurable and should be long enough to receive a downlink frame.

The second mode is optional and is called Class B [33]. While operating in this mode in addition to Class A features, sensors are capable of scheduled downlink communications. BSs occasionally transmit a time-synchronized beacon that specifies the schedule. Sensors receive these beacons and listen to the channel at the scheduled time.

The third mode is also optional and is called Class C. A Class C sensor can receive downlink frames continuously, except for times when the sensor is transmitting. Similarly to NB-Fi, Class C mode is not energy efficient, and, thus, is not often used by battery-powered devices. Therefore, we do not consider Class C sensors in this paper.

It is worth noting that Class A LoRaWAN sensors work similarly to NB-Fi end devices, operating in the DRX mode, while Class C sensors operate similarly to end devices in the CRX mode.

### 3.4. Comparison of Nominal Parameters

Table 1 lists the nominal parameters of NB-Fi, Sigfox, and LoRaWAN, which can be used to compare the considered technologies. While the considered technologies operate in the same frequency band, have the same topology, and have a similar coverage range, there are many differences between them, such as the payload size, bitrates, receiver sensitivities, etc. However, despite their differences, NB-Fi, Sigfox, and LoRaWAN networks can be configured to provide the same functionality to the user using comparable time-frequency resources, which raises the question of which technology to choose when deploying a network. To answer this question, we need to evaluate the PLR, PER, and delays for networks with many operating devices. Therefore, we consider the scenarios described in Section 4.

## 4. Scenario and Problem Statement

We consider that the server operates on the BS, and the 10,000 sensors operating in each network are distributed uniformly in a circle around the BS. NB-Fi, Sigfox, and LoRaWAN have different coverage ranges for each bitrate; however, within the 1 km range, sensors can use any bitrate. Thus, we consider a circle with a radius of 1 km. The sensors and BS are static. The LoRaWAN BS allocates two main channels and one downlink channel with 125 kHz bandwidths. In NB-Fi networks, the uplink band is 204.8 kHz wide, and the downlink band is 102.4 kHz wide; in Sigfox networks, the uplink band is 192 kHz wide. The sensors generate new frames according to an exponential distribution with a parameter λ after completing the previous transmission procedure.

All sensors use the transmission power of 25 mW. As in [34], we use the Okumura–Hata model [35] to evaluate signal propagation. We consider that the noise at the receiver has a power of −157 dBm for 50 Hz.

The frames are received successfully only if the signal-to-interference-and-noise ratio (SINR) for the transmitted frame exceeds the threshold $SINR_{min}$. For Sigfox and NB-Fi networks, $SINR_{min}$ is equal to 7 dB, where 2 dB is the BS noise factor, and 5 dB is the SNR required to achieve reliable transmission. LoRaWAN devices use the LoRa modulation [28], which requires much lower $SINR_{min}$ to successfully receive a frame. Table 4 lists $SINR_{min}$ values depending on the *SF*.

In Sigfox and NB-Fi networks, the sensors use the bitrate, assigned to them during the initialization. For Sigfox and NB-Fi, we consider a scenario where all the sensors in the network use the same bitrate. Each LoRaWAN sensor is allocated one SF during its initialization with a probability, which is inversely proportional to the duration of a frame transmitted with this *SF*. Such allocation of SFs ensures a low packet error rate that is uniformly distributed among SFs [36].

Let us consider the following three scenarios.

1. In the first scenario (further referred to as *the Unreliable Transmission of Short Packets Scenario*), the payload size of all frames is 8 bytes. NB-Fi and LoRaWAN sensors transmit frames in the unacknowledged mode. Sigfox sensors transmit each frame only once. A similar scenario is used in the case of collecting data on a daily basis when the goal is to have relevant data but not necessarily the most recent.
2. In the second scenario (further referred to as *the Unreliable Transmission of Long Packets Scenario*), the payload is generated in portions of 48 bytes, which means that LoRaWAN sensors transmit data in a single frame, while NB-Fi and Sigfox sensors operate using block transmission. To transmit 48 bytes of payload, NB-Fi sensors generate six frames, while Sigfox sensors—that can transmit up to 12 bytes in a single frame—generate four frames. Since the Sigfox specification does not mention block transmission or fragmentation, we assume that the fragmentation occurs on the application layer, which means that every frame in the block belongs to a separate transmission procedure. The time interval $T_{P}$ between procedures is uniformly distributed within the window $\left[T_{start};T_{end}\right]$. The delay between transmissions of different frames in one block in the NB-Fi network is randomized by the same rules as in Sigfox. Since the block transmission is successful only if all frames from the block are transmitted successfully, this randomization increases the probability of successful transmission.
3. In the third scenario (further referred to as *reliable transmission of short packets scenario*), the payload size is 8 bytes. However, LoRaWAN and NB-Fi sensors operate in the Class A and DRX modes, respectively, and all of the transmissions are acknowledged. Sigfox sensors transmit each frame three times and choose the interval $T_{U}$ between frame repetitions as a random value uniformly distributed within the window $\left[T_{start};T_{end}\right]$.

For the described scenarios, we state the problem to compare NB-Fi, Sigfox, and LoRaWAN networks in terms of PLR, PER, and average delay and to determine which technology is the most reliable in each scenario.

## 5. Numerical Results

We evaluate and compare the performance of NB-Fi, Sigfox, and LoRaWAN using the simulation. We have developed a discrete-event simulator, which implements the described scenarios and evaluates the dependence of PLR, PER, and average delay on the incoming rate λ for various parameters.

**Figure 10:**
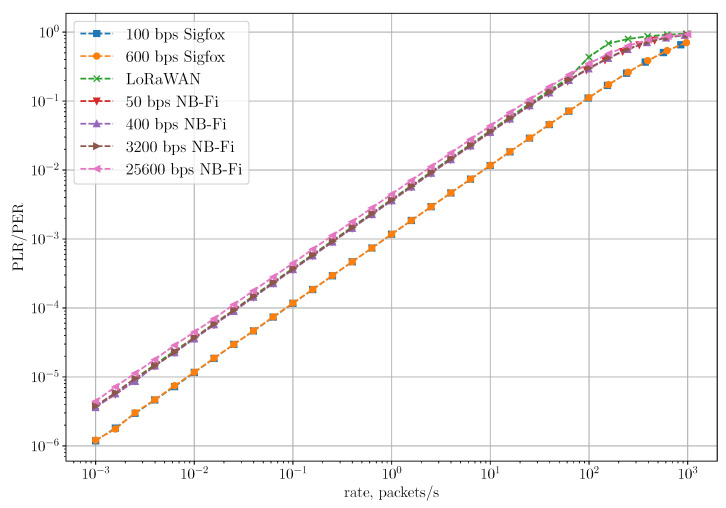
PLR and PER in the unreliable transmission of short packets scenario.

Figure 10 shows the dependence of PLR and PER on the rate for the Unreliable Transmission of Short Packets Scenario, where the rate is the ratio of the total number of generated packets during the simulation to the simulation time. In this scenario, PLR coincides with PER because frames are transmitted without repetitions or retries and can be lost only due to collisions with other frames. As we can see, in the Sigfox network, PLR and PER are lower than in NB-Fi and LoRaWAN for all rates because of different amounts of control information added in each frame in these technologies. Specifically, to transmit a 64-bit payload, Sigfox sensors transmit 157 bits, LoRaWAN sensors transmit 204 bits, and NB-Fi sensors transmit 256 bits. Therefore, Sigfox frames have the lowest overhead, which means that a frame transmission in Sigfox networks takes up fewer time-frequency resources and, thus, has a lower probability of collision than in NB-Fi and LoRaWAN networks. Note that even though overhead in LoRaWAN frames is lower than in NB-Fi frames, PLR and PER in these networks are almost the same because LoRaWAN and NB-Fi use different modulations (described in Section 3.1). On the one hand, LoRaWAN frames occupy the entire channel during the transmission, while multiple NB-Fi frames can be transmitted simultaneously without collisions on different central frequencies. On the other hand, LoRaWAN frames have lower overhead and, if transmitted with different SF, can be received and decoded simultaneously. Such differences lead to similar values of PLR and PER in LoRaWAN and NB-Fi networks.

**Figure 11:**
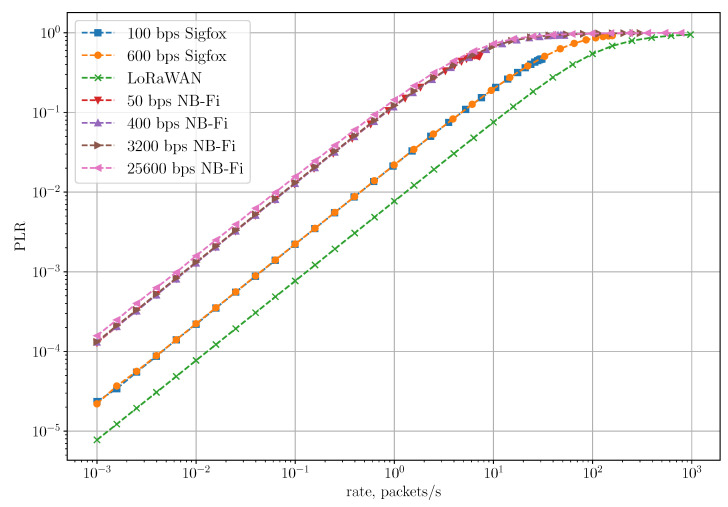
PLR for the unreliable transmission of long packets scenario.

Figure 11 shows the dependency of PLR on the rate in the Unreliable Transmission of Long Packets Scenario. The LoRaWAN network achieves the lowest PLR because of the lowest overhead. To transmit 48 bytes of payload, LoRaWAN sensors have to transmit only one frame, while Sigfox and NB-Fi sensors have to transmit 4 and 6 frames, respectively. Sigfox and NB-Fi sensors transmit frames in a block with a random delay between frames, which reduces the probability of collision. However, the PLR for block transmissions is much higher than the PLR for single frame transmission because of higher overhead in Sigfox and NB-Fi, and because losses of a single frame lead to the loss of an entire packet. Thus, LoRaWAN is more reliable compared to Sigfox and NB-Fi for transmitting long packets.

**Figure 12:**
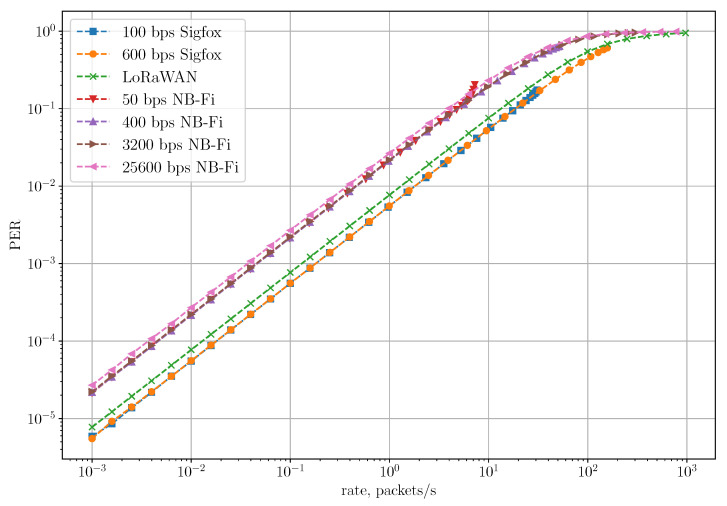
PER for the unreliable transmission of long packets scenario.

Figure 12 shows the dependency of PER on the rate in the Unreliable Transmission of Long Packets Scenario. Even though the LoRaWAN network achieves the lowest PLR, its PER is higher than in Sigfox. Since LoRaWAN sensors transmit only one frame without acknowledgments, PLR and PER for LoRaWAN coincide. At the same time, in NB-Fi and Sigfox networks, PER demonstrates how many frames in a block are received unsuccessfully, compared to PLR demonstrating the loss rate of the entire block transmission. Therefore, PER is lower than PLR for NB-Fi and Sigfox. NB-Fi networks demonstrate the highest PER due to the biggest overhead.

Note that Figure 11 shows PLR values up to some maximal rate that depends on the technology and the bitrate. For example, the 100 bps Sigfox curve stops at approximately 40 packets/s. With the considered traffic model, when sensors generate new frames only after transmission of the previous ones, at some rate, the network reaches saturation and cannot achieve higher rates: devices generate new frames at the time which is negligible compared with frame durations and inter-frame intervals. NB-Fi networks operating using 50 and 400 bps and Sigfox networks demonstrate the lowest maximal rates because the total duration of block transmission is much longer than the frame duration. Such a high ratio is the result of randomizing time interval $T_{P}$ between transmissions within a large interval because in this scenario, $T_{start}=10$ ms, and $T_{end}=100\times T_{frame}$, where $T_{frame}$ is the frame duration.

**Figure 13:**
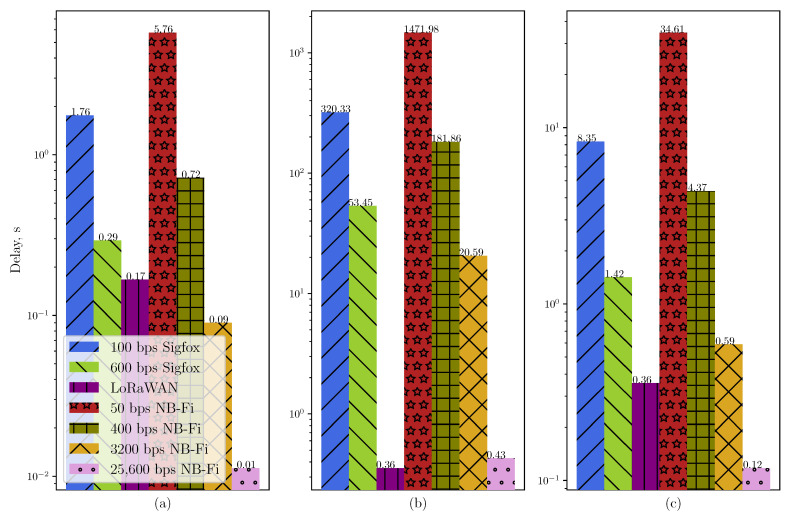
The average delay in (**a**) the unreliable transmission of short packets scenario; (**b**) the unreliable transmission of long packets scenario ($T_{start}=10$ ms, $T_{end}=100\times T_{frame}$); (**c**) the unreliable transmission of long packets scenario ($T_{start}=T_{end}=10$ ms).

Moreover, such randomization has a significant effect on the average delay. Figure 13 shows the average delay for different scenarios. The average delay for the Unreliable Transmission of Long Packets Scenario with the randomization is significantly higher than for the same scenario without the randomization. For example, NB-Fi operating at 50 bps demonstrates an average delay of almost 1500 s because the time interval between each frame is uniformly distributed within the window of [0.01;576] s, meaning a 43 times increase compared to the same scenario without randomization and 256 times increase compared with the unreliable transmission of short frames scenario. On the other hand, even with the randomization, NB-Fi at 25,600 bps demonstrates an average delay comparable with LoRaWAN networks due to a much lower frame duration. However, given that initial research showed that such randomization does not affect the PLR significantly, but notably lowers the maximal rate and increases the average delay, such randomization is unnecessary. Therefore, to consider a wider range of rates and to lower the average delay, we consider for the next scenario that $T_{start}=T_{end}=T_{G}^{min}$, where $T_{G}^{min}=10$ ms is the lowest gap duration between frames in a multiple frames U-procedure or different procedures, allowed by the Sigfox standard [21].

**Figure 14:**
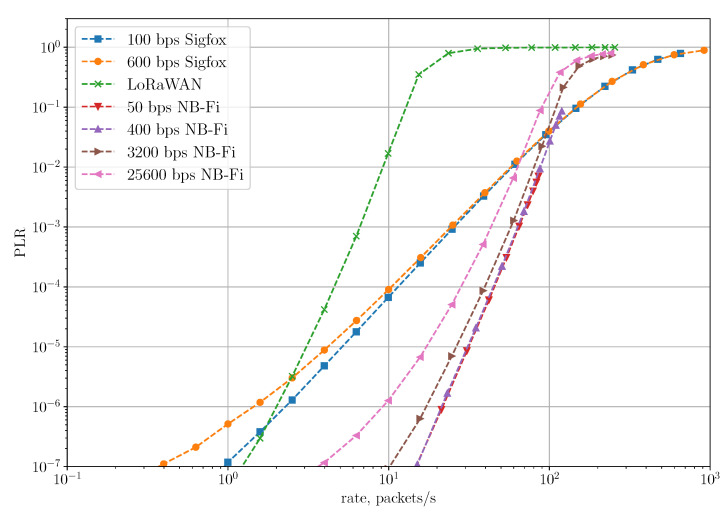
PLR for the reliable transmission of short packets scenario.

Figure 14 shows the dependence of PLR on the rate for the Reliable Transmission of Short Packets Scenario. For rates below 100 packets/s, NB-Fi networks demonstrate the lowest PLR because the duration of the receive window and time interval from which the random backoff is drawn if no ACK was received in NB-Fi are much longer than the frame duration, and since new packets are not generated until the sensor is finished the previous transmission procedure, the BS has a lot of time to transmit ACK and the channel is not overloaded with unnecessary retransmissions. In LoRaWAN networks, the durations of receive and retry windows are close to the durations of the frames, which results in redundant retransmissions, overloading the channel and, consequently, higher PLR. Sigfox sensors operate according to multiple frame U-procedure, which means that they transmit every frame thrice with the same payload. This procedure ensures reliability; however, it does not perform as well as acknowledgment mode and demonstrates higher PLR.

**Figure 15:**
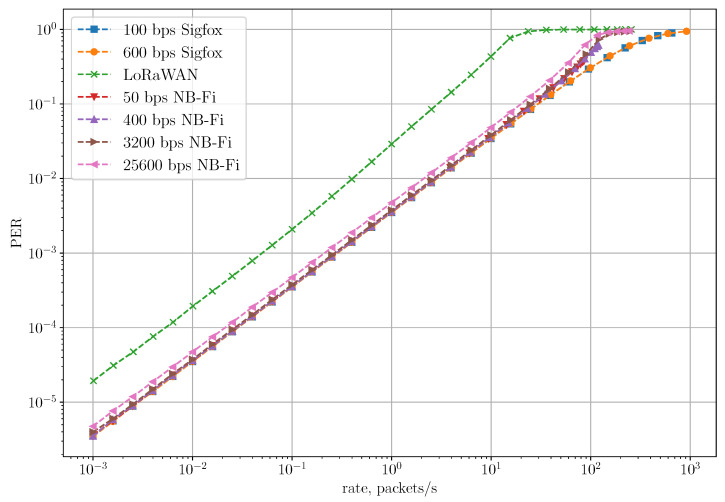
PER for the reliable transmission of short packets scenario.

Figure 15 shows the dependence of PER on the rate for the Reliable Transmission of Short Packets Scenario. PER in the LoRaWAN network is the highest compared to other considered networks for the same reason as PLR is the highest one. Sigfox and NB-Fi networks demonstrate similar PER at lower rates because of the similar channel access procedure and the same modulation in these technologies. At lower rates, PER is primarily determined by the probability of success during the first transmission attempt, which depends on the probability of frames overlapping both in time and frequency. Since in the considered scenario all sensors in Sigfox and NB-Fi networks use one bitrate, this probability does not depend on the chosen bitrate and, in our case, on the technology because frame duration is inversely proportional to the occupied frequency band. However, at higher rates, NB-Fi provides higher PER than Sigfox because of the frequency selection algorithm used in NB-Fi for downlink transmission. As was explained in Section 3.3, for downlink transmissions, the server calculates the central frequency as a function of bitrate and Modem_ID, which means a higher probability that the server chooses the same central frequencies for multiple sensors at higher rates. Thus, ACK transmission is delayed and if this delay exceeds the timeout duration ($T_{L}$ in Table 3) the ACK is not transmitted. On the other hand, in Sigfox networks, frames are not acknowledged, therefore resulting in lower PER at higher rates.

Note that the curves in Figure 14 and Figure 15 stop at some maximal rate. NB-Fi networks, where sensors operate using bitrates of 50 and 400 bps, demonstrate the lowest maximal rates. Specifically, the rate does not achieve higher values than approximately 80 packets/s for 50 bps and 120 packets/s for 400 bps because at such rates the devices operate in saturation: they generate new frames after a delay that is much lower than the total duration of data frames, receive windows, and the window from which the random delay is drawn. At the same time, NB-Fi networks that use bitrates of 3200 and 25,600 bps can generate more packets and thus achieve higher rates. In addition, at high rates, NB-Fi networks that use 3200 or 25,600 bps bitrates demonstrate higher PLR than Sigfox networks because of the high probability of overlap in frequency and a relatively small ratio of the receive and retry durations to the frame duration.

**Figure 16:**
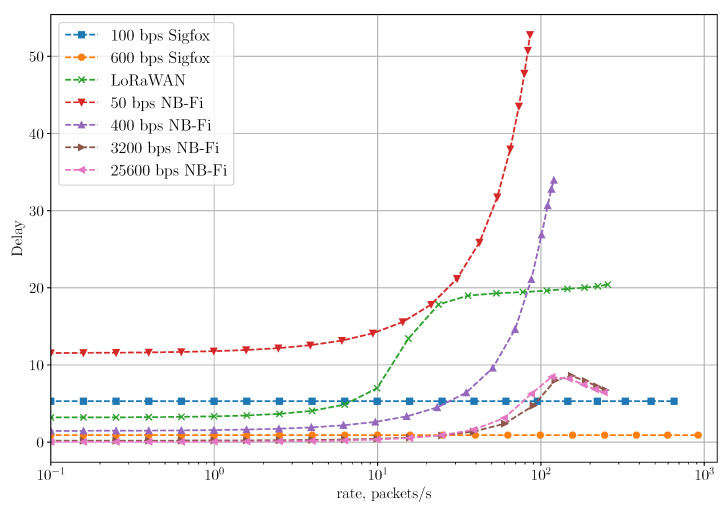
The average delay for the reliable transmission of short packets scenario.

Figure 16 shows the average delay for the Reliable Transmission of Short Packets Scenario. Since Sigfox networks operate without acknowledgments, the average delay is constant for any rate and equals $3\times T_{frame}+2\times T_{G}^{min}$. In NB-Fi and LoRaWAN networks, at lower rates, the average delay is primarily determined by the round-trip time, which is inversely proportional to the bitrate. When the rate increases, so does the number of retries required for the successful frame transmission, which significantly increases the average delay. In LoRaWAN networks, the average delay starts increasing more rapidly and at lower rates than in NB-Fi networks because of the collision avalanche caused by an inefficient collision resolution approach [37]. However, the increase rate significantly lowers at approximately 200 packets/s, which correlates with PLR reaching a plateau at the same rates. In NB-Fi networks operating at 3200 and 25,600 bps, the average delay decreases after reaching the maximal value because, at such a rate, the PLR is high and frames are either lost and do not affect the average delay or are transmitted successfully without many retries.

Let us conclude the numerical results. Even though the considered technologies have similarities, such as, for example, channel access in NB-Fi and Sigfox, and acknowledgment mode in NB-Fi and LoRaWAN, the technologies perform best in different scenarios. Sigfox is the most reliable in the scenario where only short packets are transmitted in an unreliable way. LoRaWAN performs better if only long packets are transmitted in a network, while NB-Fi is more suitable if short packets have to be transmitted most reliably.

## 6. Conclusions

This is the first paper that compares NB-Fi, Sigfox, and LoRaWAN not just by their nominal operational parameters but also by PLR, PER, and delays in different scenarios. In this paper, we have compared and analyzed such LPWAN technologies as Sigfox, LoRaWAN, and NB-Fi in three scenarios: the unreliable transmission of short packets, the unreliable transmission of long packets, and the reliable transmission of short packets. Using the simulation, we have demonstrated and analyzed the average delay and the dependence of PLR, PER, and delays on the rate. The analysis has revealed that Sigfox, LoRaWAN, and NB-Fi perform best in different scenarios. Sigfox is the most reliable if all of the transmitted packets are short and sent in the unacknowledged mode only once. If sensors transmit only long packets in an unreliable way, LoRaWAN performs better than NB-Fi and Sigfox. NB-Fi demonstrates the highest reliability if sensors transmit short packets in acknowledgment mode or according to multiple frame procedure. Therefore, the considered technologies have their advantages and disadvantages, which manifest themselves in different scenarios. The results obtained in this paper should be taken into account when selecting an appropriate LPWAN technology for a specific IoT application.

## Figures and Tables

**Table 1:** Comparison of nominal properties of the considered technologies.

	NB-Fi	Sigfox	LoRaWAN
Frequency Band	Sub-GHz ISM	Sub-GHz ISM	Sub-GHz ISM
Topology	star of stars	star of stars	star of stars
Bitrate, bps	(a) 50; (b) 400; (c) 3200; (d) 25,600.	(a) 100; (b) 600.	(a) 250; (b) 440; (c) 980; (d) 1760; (e) 3125; (f) 5470.
BW, kHz	UL: ≥51.2	192	125, 250, 500
	DL: ≥102.4		
PL size, bytes	8	≤12	(a) ≤51; (b) ≤51; (c) ≤51; (d) ≤115; (e) ≤242; (f) ≤242.
Modulation	UL: DBPSK	UL: DBPSK	LoRa
	DL: DBPSK, BPSK	DL: GFSK	
$S_{crit}$, dBm	(a) −150; (b) −141; (c) −132; (d) −123.	UL: −135; DL: −126	(a) −137; (b) −136; (c) −134; (d) −131; (e) −128; (f) −125.
Operating	NRX, DRX, CRX	U-, B- procedure	Class A, B, C
modes		S-, M- frames ^1^	
ACK mode	+	–	+
Block	+	+	not considered
transmission			
Coverage range,	urban: 10	urban: 10	urban: 5
km [20,25,26]	rural: 40	rural: 40	rural: 20
	UL: Convolutional or	UL: Convolutional	
Encoding	non-systematic polar	DL: BCH15-11	Hamming
	DL: Zigzag		
Code rates	UL: 5/8	1/3	4/5,4/6,4/7,4/8
	DL: 1/2		
Encryption	Magma	AES-128	AES-128
[21,22,24]			

^1^ single, multiple frames transmission.

**Table 2:** Frame parameters in LoRaWAN.

*SF*	$T_{\mathrm{up}}^{\max}$, s	$T_{\mathrm{up}}^{\min}$, s	$T_{\mathrm{down}}^{\max}$, s	$T_{\mathrm{down}}^{\min}$, s	PL, Bytes
12	3.121	1.155	3.072	1.057	51
11	1.708	0.602	1.683	0.553	51
10	0.688	0.268	0.678	0.258	51
9	0.667	0.144	0.656	0.134	115
8	0.697	0.077	0.692	0.072	242
7	0.396	0.0425	0.393	0.039	242

**Table 3:** Time interval values in NB-Fi.

Bitrate, bps	$T_{f}$, ms	$T_{B}$, ms	$T_{L}$, ms	$T_{\mathrm{rnd}}$, ms
50	5760	5900	60,000	5000
400	720	740	30,000	1000
3200	90	95	6000	100
25,600	11.25	15	6000	100

**Table 4:** Minimal SINR required at the LoRaWAN receiver input.

*SF*	${\mathrm{SINR}}_{\min}$, dB
12	–20
11	–17.5
10	–15
9	–12.5
8	–10
7	–7.5

## Data Availability

Not applicable.

## Abbreviations

The following abbreviations are used in this manuscript:

ACK	Acknowledgement
AES	Advanced Encryption Standard
B-procedure	Bidirectional procedure
BCH	Bose–Chaudhuri–Hocquenghem code
BPSK	Binary phase-shift keying
BS	Base Station
CRC	Cyclic redundancy check
CRX	Continuous RX
DBPSK	Differential binary phase-shift keying
DL	Downlink
DRX	Discontinuous RX
ECC	Error Correction Code
FT	Frame Type
GFSK	Gaussian frequency shift keying
IIoT	Industrial IoT
IoT	Internet of Things
LPWAN	Low-power wide-area network
MAC	Medium access control
NRX	No RX
PDR	Packet Delivery Rate
PER	Packet Error Rate
PHY	Physical layer
PLR	Packet Loss Rate
RSSI	Received Signal Strength Indicator
RX	Receiver
*SF*	Spreading Factor
SINR	Signal to interference and noise ratio
SNR	Signal-to-noise ratio
U-procedure	Uplink procedure
UL	Uplink
